# Comparative Analysis of *Lactobacillus gasseri* and *Lactobacillus crispatus* Isolated From Human Urogenital and Gastrointestinal Tracts

**DOI:** 10.3389/fmicb.2019.03146

**Published:** 2020-01-22

**Authors:** Meichen Pan, Claudio Hidalgo-Cantabrana, Yong Jun Goh, Rosemary Sanozky-Dawes, Rodolphe Barrangou

**Affiliations:** Department of Food, Bioprocessing and Nutrition Sciences, North Carolina State University, Raleigh, NC, United States

**Keywords:** probiotics, niche-specific adaptation, *Lactobacillus*, women’s health, *in vitro* vaginal fluid model

## Abstract

*Lactobacillus crispatus* and *Lactobacillus gasseri* are two of the main *Lactobacillus* species found in the healthy vaginal microbiome and have also previously been identified and isolated from the human gastrointestinal (GI) tract. These two ecological niches are fundamentally different, notably with regards to the epithelial cell type, nutrient availability, environmental conditions, pH, and microbiome composition. Given the dramatic differences between these two environments, we characterized strains within the same *Lactobacillus* species isolated from either the vaginal or intestinal tract to assess whether they are phenotypically and genetically different. We compared the genomes of the *Lactobacillus* strains selected in this study for genetic features of interest, and performed a series of comparative phenotypic assays including small intestinal juice and acid resistance, carbohydrate fermentation profiles, lactic acid production, and host interaction with intestinal Caco-2 and vaginal VK2 cell lines. We also developed a simulated vaginal fluid (SVF) to study bacterial growth in a proxy vaginal environment and conducted differential transcriptomic analysis between SVF and standard laboratory MRS medium. Overall, our results show that although strain-specific variation is observed, some phenotypic differences seem associated with the isolation source. We encourage future probiotic formulation to include isolation source and take into consideration genetic and phenotypic features for use at various body sites.

## Introduction

Recent advances in human microbiome research have revealed that bacterial strains of the same species can be isolated from different body sites such as the gastrointestinal tract and vaginal tract. These two human body sites are the main targets for probiotic applications. However, it remains unclear whether tailored probiotic formulations should be considered for specific applications to enhance probiotic efficacy. The results of this research reveal strain-dependent variation, and niche-specific adaptation to intestinal vs. vaginal environments. Overall, this work demonstrated that the probiotic functionality can vary with isolation source, and should be taken into consideration during probiotic formulation for enhanced efficacy.

Significant variations in microbial diversity and abundance between individuals and body sites have been revealed by the Human Microbiome Project (Human Microbiome Project Consortium, 2012; Lozupone et al., 2012). The human gastrointestinal (GI) tract represents one of the most complex microbial communities that have ever been studied, composed of highly diverse microbial groups such as *Bacteroides*, *Lactobacillus*, *Escherichia*, *Clostridium*, and *Bifidobacterium* (Arumugam et al., 2011; Lozupone et al., 2012). In contrast to the complex gut microbiome, vaginal microbiome is featured with low bacterial diversity, variation among different ethnicity groups, and commonly dominated by *Lactobacillus* species such as *Lactobacillus crispatus*, *Lactobacillus gasseri*, *Lactobacillus jensenii*, and *Lactobacillus iners* (Ravel et al., 2011). Besides the different microbiome composition, the two ecological niches, human GI tract and vaginal tract, also differ significantly in terms of their epithelial cell lining, nutrient availability, and pH. The human vaginal tract, lined with multiple layers of stratified squamous epithelial cells (Anderson et al., 2014) and heavily regulated by the fluctuation of estrogen hormones (Farage and Maibach, 2006), is acidified to pH 3.5–4.5 mainly by lactic acid produced by the commensal microbiota (Boskey et al., 2001). With secretion of α-amylase from the host, the commensal bacteria in the vaginal tract are presumably able to utilize glycogen as a carbon source, the main carbohydrate stored in the superficial and intermediate layer of the epithelial cells and released when epithelial cells die and slough off (Anderson et al., 2014; Spear et al., 2014; van der Veer et al., 2019). On the other hand, the human GI tract is comprised of a single layer of columnar epithelial cells coated by a thick layer of mucus secreted by goblet cells (Turner, 2009). Depending on the section of the human intestine, it has blends of gastric juice with various mixtures of digestive enzymes and bile salts, which are absent from the vaginal tract (Ianiro et al., 2016). The pH in the GI tract ranges from pH 2 in the stomach to pH 7.4 in the terminal ileum (Fallingborg, 1999).

More extensive investigation of the human microbiome has further elicited interest in using probiotic bacteria, defined as “live microorganisms that, when administered in adequate amounts, confer a health benefit on the host” (Hill et al., 2014), to restore the native microbiota, especially the gut microbiota, to alleviate dysbiosis (Hibberd et al., 2017; Daliri et al., 2018). *L. crispatus* and *L. gasseri* are commensal bacteria found in both human GI tract (Walter, 2008) and lower female urogenital tract. Their implication on women’s health have been explored in the recent years (Stapleton et al., 2011; Matsuda et al., 2018; Phukan et al., 2018; Takada et al., 2018). Preliminary clinical trials have shown that intro-vaginal probiotic treatment with *L. crispatus* (intravaginal suppository probiotic Lactin-V; Osel) could significantly lower the reoccurrence rate of urinary tract infections in premenopausal women (Stapleton et al., 2011). *L. crispatus* JCM 7696 has also been shown to accelerate wound healing in vaginal epithelial cell line (Takada et al., 2018). *L. gasseri* American Type Culture Collection (ATCC) 9857 has been found to significantly inhibit the adhesion of *Trichomonas vaginalis* to human vaginal ectocervical cells (Phukan et al., 2018). Supernatant of *L. crispatus* JCM 1185 and *L. gasseri* JCM 1131 significantly inhibited the colonization of *Candida albicans* by reducing the expression of biofilm formation-related genes (Matsuda et al., 2018). It is recognized that bacteria residing in different environments could develop niche-specific adaptation, providing competitive advantages for colonizing each niche (Oh et al., 2010; Kim et al., 2016). O’Flaherty et al. (2018) reported that lactobacilli containing bile salt hydrolase (BSH) proteins are usually associated with vertebrate-adapted lifestyle rather than environmental and plant-associated lifestyle. Possible mechanism for *L. crispatus* to dominate vaginal tract has been suggested as the presence of glycogen degradation genes in their genomes (van der Veer et al., 2019). Others have investigated if specific proteins exist among vaginal species allowing vaginal specialization (Mendes-Soares et al., 2014). Taken together, it seems that strains within the same species that adapted to different environments might develop distinct phenotypes and genotypes that distinguish them. Such knowledge would be especially helpful and critical in terms of probiotic formulation.

In this study, we aimed to investigate the phenotypic and genotypic diversity and potential niche-specific adaption of *L. gasseri* and *L. crispatus* strains isolated from human intestinal and vaginal tracts. We first performed a comparative genomic analysis of the *Lactobacillus* strains in this study. We then characterized these strains through a series of phenotypic assays such as carbohydrate metabolism, acid resistance, small intestine juice exposure, and interaction with intestinal and vaginal epithelial cells. We also performed differential transcriptomic analysis in simulated vaginal fluid (SVF) vs. MRS to examine the transcriptional response of the strains under growth environment resembling the vaginal conditions. Our results suggest that although some phenotypic differences are strain-dependent, isolation source-dependent characteristics likely reflect niche-specific adaption. These findings should be taken into account for rational probiotic formulations for enhanced and body-site specific applications.

## Materials and Methods

### Bacteria Strains and Growth Conditions

The *Lactobacillus* strains used in this study are listed in Table 1. *L. crispatus* JV-V01 (Lcr_V) and *L. gasseri* JV-V03 (Lga_V) were isolated from the female urogenital tract. *L. crispatus* NCK1350 (Lcr_I) and *L. gasseri* NCK1347 (Lga_I) were isolated from healthy human endoscopies. Lactobacilli were grown from −80°C glycerol stock in de Man-Rogosa-Sharpe (MRS) broth (Difco Laboratories, Detroit, MI, United States) at 37°C in an anaerobic chamber overnight, followed by 1% (v/v) subculture in fresh MRS to prepare working culture for assays and analyses. Lcr_V and Lga_V were obtained from BEI collection. Lcr_I and Lga_I were from our laboratory’s culture collection, isolated from endoscopy samples of probiotic study at the Thomas Jefferson University, PA, United States, in 1997.

**TABLE 1:** Strains included in this study.

Species	Strain	References	Isolation source	Designation	Accession number
*Lactobacillus crispatus*	JV-V01	Witkin et al., 2014	Female urogenital tract	Lcr_V	ACKR00000000
	NCK1350	Hidalgo-Cantabrana et al., 2019	Human endoscopy	Lcr_I	SGWL00000000
*Lactobacillus gasseri*	JV-V03	Stout et al., 2018	Female urogenital tract	Lga_V	ACGO00000000
	NCK1347	Stout et al., 2018	Human endoscopy	Lga_I	CP043924

### Comparative Genomic Analysis

Bacterial genomes for Lcr_V and Lga_V were obtained from NCBI. Lcr_I was previously sequenced in our lab and its genome was available from NCBI. The genome accession numbers are listed in Table 1. For the genome of Lga_I, DNA preparation, genome sequencing, and assemblies were performed at University of Illinois at Urbana-Champaign (IL, United States). DNA was extracted from overnight 37°C MRS with MasterPure Gram positive DNA purification kit (Lucigen, WI, United States) The library was prepared with the Hyper Library construction kit from Kapa Biosystems (Roche). The library was sequenced on MiSeq flow cell for 251 cycles from each end of the fragments (paired-reads of 250 nt in length) using a MiSeq 500-cycle sequencing kit version 2. Porechop was used to remove adaptors sequence and remove reads <1,000 bp before assembly with Unicycler assembler (v0.4.4).

The four bacterial genomes were then annotated using Prokka v1.13.3 (Seemann, 2014) and the downstream sequence analyses were performed on gene features potentially important for probiotic functionality. Genomic comparison between strains was done using Roary v3.12.0 (Page et al., 2015) where the predicted open reading frames (ORFs) of each genome were analyzed by BLAST to identify common and unique genes (minimum percentage nucleotide identity of 95%). Functional classification of genes was performed using EggNOG 5.0 (Huerta-Cepas et al., 2019). Surface proteins were predicted using SignalP−5.0 (Armenteros et al., 2019). The nucleotide coding sequence of the predicted proteins were then analyzed by BLAST within the same species to identify unique and common surface protein (minimum percentage nucleotide identity of 95%). Exopolysaccharides (EPS) gene cluster was annotated by blasting against EPS cluster manually identified in Lcr_I genome with a threshold of 40% nucleotide identity. The selected genes were then manually curated. Prediction of prophage regions was carried out using PHASTER (Arndt et al., 2016). Clustered regularly interspaced short palindromic repeat (CRISPR) and CRISPR-associated protein (Cas) system was detected using CRISPRdisco (Crawley et al., 2018). The spacer-repeat arrays, the Cas proteins and neighboring proteins were extracted manually. Carbohydrate metabolism operons were annotated using BLAST search with known carbohydrate metabolism operons in *Lactobacillus acidophilus* NCFM (with a threshold of 40% nucleotide identity). The annotated operons were then manually curated.

### Simulated Small Intestinal Juice Assay

A 0.5% NaCl solution mixed with 3 g/L oxgall (Difco Laboratories, Detroit, MI, United States) was prepared and adjusted to pH 8.0 with 0.1 M NaOH as needed. The solution was then autoclaved and stored at 4°C. On the day of the experiment, the simulated small intestinal juice was prepared by adding 1 mg of pancreatin (MP Biomedicals, Solon, OH, United States) to 1 ml of the NaCl/oxgall solution (the final concentration of pancreatin was 0.83 mg/ml) and pre-warmed at 37°C prior to the experiment. Stationary phase cultures grown in MRS broth for 16 h (1 ml) was centrifuged at 9,600 × *g* for 1 min in sterile 1.5 ml microcentrifuge tubes in a tabletop centrifuge. Each cell pellet was washed twice with 1 ml of PBS and resuspended in 1 ml of PBS. An aliquot of the cell suspension (0.2 ml) was mixed with 1 ml of freshly prepared pre-warmed simulated small intestinal juice as described above. Cell suspensions were incubated at 37°C anaerobically and viable cells were enumerated on MRS agar plates every hour for 4 h.

### Acid Challenge

Stationary phase (18 h overnight culture) or log phase (OD_600_ = 1.0) cultures (5 ml) grown in MRS at 37°C anaerobically were centrifuged at 3,220 × *g* for 10 min (Eppendorf, Centrifuge 5810R) at room temperature and washed twice with PBS. Each cell pellet was then resuspended in 5 ml of MRS or MRS adjusted with D/L-lactic acid (Sigma Aldrich, St. Louis, MO, United States) or HCl to a final pH of 4. The cell suspension was immediately plated on MRS agar for time 0 h count. The bacterial suspension was then incubated at 37°C anaerobically, and the viable cells were enumerated on MRS agar plates at 2, 4, and 18 h.

### Lactic Acid Production

The lactic acid concentration in 18 h culture supernatant was measured by EnzyChrom^TM^ L-Lactate Assay Kit, and EnzyChrom^TM^ D-Lactate Assay Kit following the manufacturer’s protocol. The optical density at 565 nm at time 0 and at 20 min was recorded. The interpolation of sample OD in the standard curve was used to determine the lactate concentration for each lactate isomer.

### Carbohydrate Utilization Profiles

Carbohydrate utilization was initially analyzed by API test (BioMerieux, Inc., Marcy l’Etoile, France) following the manufacturer’s instructions. Based on results from API test, growth experiments on selected carbohydrate substrates in semi-defined medium (SDM) (Kimmel and Roberts, 1998) were conducted. For this purpose, overnight cultures grown in MRS medium were washed with PBS twice and resuspended in SDM basal medium without carbohydrates. The SDM medium was supplemented with 1% (w/v) of various sugars and 0.05% (w/v) L-cysteine (Fisher Scientific) to achieve anaerobic condition, and inoculated with 1% of the culture suspension. Three technical replicates were included in each of the three biological replicates. The 96-well microplate (Corning Costar, Corning, NY, United States) was sealed with clear adhesive film, and OD_600_ measurement was measured hourly for 46 h at 37°C in a Fluostar Optima microplate reader (BMG Labtech, Cary, NC, United States).

### Caco-2 and VK2 Cell Cultures

The Caco-2 and VK2 cell lines were purchased from the ATCC. Caco-2 cells were cultured in minimum essential medium (MEM) containing Earle’s salts and 2 mM L-glutamine supplemented with 10% FBS (Gibco), MEM non-essential amino acids (Gibco), MEM sodium pyruvate (Gibco), and antibiotic/antimycotic solution (Gibco). VK2/E6E7 human vaginal epithelial cells were cultured in keratinocyte-serum free medium (Gibco) supplemented with 0.05 mg/ml bovine pituitary extract, 0.1 ng/ml human recombinant EGF, 0.4 mM calcium chloride, penicillin, and streptomycin, as described previously (Fichorova et al., 1997). For both cell lines, the cells were split every 3–4 days before reaching confluence and the cell culture medium was changed every 48 h. Both cell lines were cultured at 37°C with 5% CO_2_ humidified atmosphere in a 75 cm^2^ cell culture flask. Cells were allowed to reach confluence and differentiate for 21 days in a 12-well plate or a 12-well Transwell plate (Corning) before experiments.

### *In vitro* Epithelial Adhesion Assay

To prepare for adhesion assays, Caco-2 cells or VK2 cells were seeded in cell culture treated 12-well plates at a seeding density of 1.6 × 10^5^ cells/well or 5 × 10^4^ cells/well in a total volume of 2 ml, respectively. On the day of the experiment, the confluent and fully differentiated cell monolayers were rinsed with PBS buffer twice and antibiotic/antimyotic-free cell culture medium was added. Overnight bacterial cultures grown in MRS medium were washed twice in PBS and resuspended in antibiotic/antimyotic free cell culture medium. The OD_600_ was adjusted to reach MOI 25 (the ratio of bacterial cell to epithelial cell was 25:1). The cell suspension was diluted and enumerated on MRS agar plates to calculate the amount of bacteria added. Bacteria were incubated with the cell monolayers for 1 h at 37°C under 5% CO_2_ humidified atmosphere followed by washing five times with PBS to remove unadhered bacterial cells. The cell monolayer was then incubated with 1 ml of 0.05% (v/v) Triton ×-100 (Acros, United States) per well for 10 min. The epithelial cells were then scraped off and transferred to a 5 ml tube. The cell-Triton mixture was then vortexed vigorously to completely disintegrate the cell monolayer. The cell suspensions were diluted and enumerated on MRS agar plates to calculate the percentage of adhesion.

### Transwell Assay

Caco-2 cells or VK2 cells were seeded in Transwell plates (Corning, 12-mm diameter, polyester, 0.4 μm pore size) at a seeding density of 3 × 10^5^ cells/insert with 0.5 ml of media in the apical chamber and 1.5 ml of media in the basolateral chamber. The trans-epithelial electrical resistance (TEER) was measured using an epithelial voltohmmeter (EVOM^2^) (World Precision Instruments, Sarasota, FL, United States). On the day of the experiment, the confluent and fully differentiated cells were rinsed with PBS buffer twice and antibiotic/antimyotic-free cell culture medium was added. Overnight bacteria cultures grown in MRS medium were washed in PBS twice and resuspended in antibiotic/antimyotic-free cell culture medium. The OD_600_ was adjusted to reach MOI 10. The cell suspension was enumerated to calculate the amount of bacteria added. Bacteria were incubated with the cell line for 24 h at 37°C with 5% CO_2_ humidified atmosphere. TEER was tested before and after incubation. Transwells with only cell culture mediumor with cell line and medium were included as blank and control, respectively. The TEER was calculated as (R_total_−R_blank_) multiplied by membrane area.

### Scanning Electron Microscopy (SEM)

The SEM sample preparation and imaging were performed at the Center for Electron Microscopy at North Carolina State University, Raleigh, NC, United States. Bacteria were centrifuged for 10 min at 2,500 rpm, the supernatant removed and resuspended in 10 ml of 3% glutaraldehyde in 0.1 M Na cacodylate buffer pH 5.5 and stored at 4°C until processed. Bacterial suspensions were filtered using a 0.4 μM pore polycarbonate Nucleopore filter. Filters containing bacteria were washed with three 30-min changes of 0.1 M Na cacodylate buffer pH 5.5 and then dehydrated with a graded series of ethanol to 100% ethanol and were critical point dried (Tousimis Samdri-795, Tousimis Research Corp., Rockville, MD, United States) in liquid CO_2_. Dried filters were mounted on stubs with double-stick tape and silver paint and sputter coated (Hummer 6.2 sputtering system, Anatech, Union City, CA, United States) with 50Å Au/Pd. Samples were held in a vacuum desiccator until viewed using a JEOL JSM-5900LV SEM (JEOL, Peabody, MA, United States). Images were acquired at a resolution of 1280 × 960 pixels.

### Preparation of Simulated Vaginal Fluid (SVF) and Growth Experiments

The SVF was prepared according to the formulation shown in Table 2. To prepare 500 ml of SVF, part A ingredients were mixed together in 440 ml water with 3.31 ml of DL-lactic acid (88 mM final concentration) (Sigma Aldrich, St. Louis, MO, United States). The acidified Part A mixture was then filter-sterilized using a 0.45 μM filter (Thermo Fisher Scientific, Fair Lawn, NJ, United States). Each component of Part B was prepared and filter-sterilized using a 0.45 μM filter separately due to low solubility. Albumin solution 4% (w/v) (bovine serum) was prepared by heating the suspension to increase solubility. After filter sterilization, 5 ml of 2.5% (100×) mucin solution (porcine stomach), 50 ml of 4% (100×) albumin solution and 5 ml of 100× vitamin solution were added to the sterile acidified Part A mixture. The SVF was then mixed and a 5 ml sample was taken to confirm the final pH at 4.5. Fresh SVF was prepared for each experiment, and stored at 4°C. The α-amylase was added to SVF right before the experiment to reach a final concentration of 2 mU/ml. The α-amylase stock solution (10 Unit/ml) was prepared in Tris–HCl (pH 7.5) solution and filter sterilized and stored at −20°C. All reagents were purchased from Sigma Aldrich (St. Louis, MO, United States).

**TABLE 2:** Formulation of Simulated Vaginal Fluid (SVF).

Components	Ingredients	Final Concentration (w/v)
Part A	Tween 80	0.1%
	Ammonium citrate	0.2%
	Sodium acetate	0.5%
	MgSO_4_-7H_2_O	0.01%
	MnSO_4_-H_2_O	0.005%
	K_2_HPO_4_	0.2%
	No.3 proteose peptone	0.3%
	Urea	0.05%
	Glucose	1%
	Glycogen	1%
	Lactic acid	88 mM
Part B	Mucin	0.025%
	Albumin	0.4%
	Vitamin solution	1×
	α-amylase	2 mU/ml

For growth experiments, overnight bacterial cultures grown in MRS were washed twice with phosphate-buffered saline (PBS, ph7.4: gibco) and inoculated at 1% (v/v) into SVF or acidified MRS medium at pH 4.5 (adjusted with lactic acid) with 0.05% (w/v) L-cysteine. Growth in SVF or acidified MRS was monitored in sealed 96-well microplates (Corning Costar, Corning, NY, United States) at 37°C in a Fluostar Optima microplate reader (BMG Labtech Cary, NC, United States). The optical density at OD_600_ was measured every hour for 25 h. Similarly, the strains were grown in the acidified MRS broth at pH 4.5 (adjusted with lactic acid with 0.05% (w/v) cysteine supplementation). Growth experiments were performed in biological triplicates.

### RNA-Seq and Transcriptomic Analysis

Total RNA from mid-log phase bacterial cells in MRS or SVF was extracted and prepared as described previously (Johnson et al., 2016; Crawley and Barrangou, 2018). The RNA-seq data has been deposited in the NCBI genome database under the BioProject ID PRJNA546252. The accession numbers are SAMN11958879 to SAMN11958894. Briefly, bacterial cultures were grown to an OD_600_ of 0.6 in either MRS or SVF medium at 37°C anaerobically. Total RNA was isolated using the Zymo Direct-zol RNA MiniPrep kit (Zymo Research, Irvine, CA, United States) followed by DNase treatment and quality checked using an Agilent 2100 Bioanalyzer (Agilent Technologies, Santa Clara, CA, United States). RNA library preparation and sequencing were carried out by the High-throughput Sequencing and Genotyping Unit of the Roy J. Carver Biotechnology Center, University of Illinois at Urbana-Champaign, IL, United States. The Ribo-Zero bacterial kit (Illumina, San Diego, CA, United States) was used to remove rRNA from each sample. TruSeq stranded RNA sample preparation kit (Illumina) was used for library preparation. The quality of raw sequencing reads was checked by FastQC version 0.11.8^1^. The resulting reads were mapped to individual bacterial genomes using Geneious Mapper with default setting in Geneious v11.1.5 (Kearse et al., 2012). Differential transcriptomic analyses between MRS and SVF were performed using DESeq2 in Geneious 11.1.5. Transcriptional analysis was based on normalized transcripts per million (TPM) and fold-change in expression between two conditions (Log2 differential expression ratio). Cluster of Orthologous Groups (COGs) analysis was performed using eggNOG version 5.0 (Huerta-Cepas et al., 2019). Common upregulated genes in SVF across the four *Lactobacillus* strains were determined by protein orthologs analysis using Roary (40% nucleotide identity used as the threshold) (Page et al., 2015).

### Statistical Analysis

For all growth curves, bar graphs and line graphs, three biological replicates (either two or three technical replicates within each biological replicated) were conducted and included for statistical analysis. All time points in growth curves were presented as the average of the three biological replicates with average errors <7%. The error bars in the bar graphs and line graphs represent the standard deviation. The significant test was analyzed using Welch’s *t* test, comparing the two unpaired groups with the null hypothesis that the two groups shared the same average. For the TEER analyses, one-way ANOVA and Tukey test were performed to see if the means were significantly different among groups. ^∗^*p*-value < 0.05, ^∗∗^*p*-value < 0.01, ^∗∗∗∗^*p*-value < 0.0001. The statistical analyses were performed in R studio, v1.2.5001.

## Results

### Genomic Comparative Analysis in Features of Interest

**FIGURE 1:**
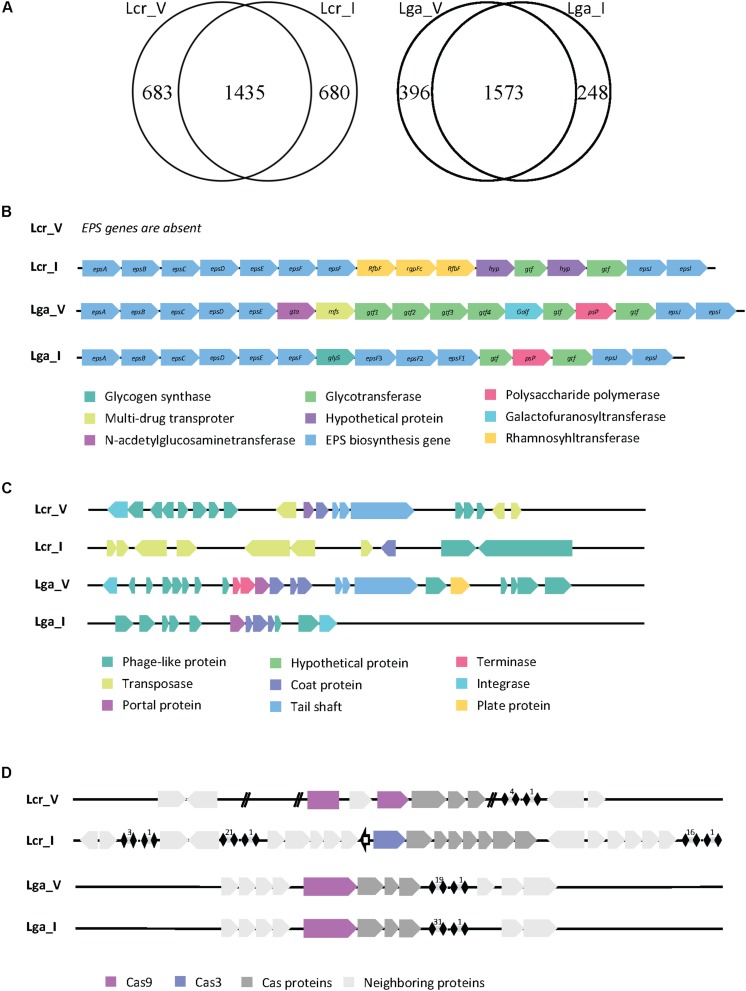
Comparative genomic analysis of *Lactobacillus* strains. Genome comparison between strains in *Lactobacillus crispatus* or *Lactobacillus gasseri* revealed unique and common genes within each species **(A)**. The EPS cluster genes were identified in each genome except Lcr_V **(B)**. The prophage regions were identified, and the general gene function was annotated **(C)**. The CRISPR-Cas system was identified in each genome **(D)**.

The genome size was similar across all four strains. *L. crispatus* strains (2.3 Mbp for Lcr_V, 2.1 Mbp for Lcr_I) have slightly higher genome sizes than *L. gasseri* strains (2 Mbp for Lga_V, 1.9 Mbp for Lga_I). The larger genome size of *L. crispatus* corresponds to a higher number of coding sequence (2118 for Lcr_V and 2115 for Lcr_I) compared to *L. gasseri* strains (1933 for Lga_V and 1785 for Lga_I) (Figure 1A). *L. crispatus* strains also shared a higher GC content (37.2% for Lcr_V, 37% for Lcr_I) than *L. gasseri* strains (34.6% for Lga_V, 35% for Lga_I).

Given the vast differences between the vaginal and intestinal environment, we are interested to investigate how surface proteins of the four *Lactobacillus* strains differ, since bacterial surface mediates direct host-microbe interaction. We compared the surface proteins among strains using Signal-Pv5.0 (Armenteros et al., 2019) that detects signal peptides and thus captures proteins being secreted outside or associated to the cell membrane. This information is provided in the Supplementary File S1, where the unique surface proteins in each species were highlighted in orange. The signal peptide cleavage site position was also predicted when appropriate and reported. Two main kinds of signal peptides were found in the *in silico* search. One was signal peptide secreted in general secretory pathway and cleaved by signal peptidase I (Sec/SPI). The other one was signal peptide associated with lipoproteins and cleaved by signal peptidase II (Sec/SPII). There were 48 out of 189, 46 out of 193 surface protein identified as unique to Lcr_V and Lcr_I, respectively. Some common proteins shared within *L. crispatus* strains are S-layer protein, membrane carbohydrate and ions transport proteins, and hypothetical proteins with unknown function. Some of the unique proteins present in Lcr_V are multidrug resistance protein MdtG, sensor histidine kinase RcsC, and a phosphate-binding protein PstS 1. Both Lcr_V and Lcr_I had the glycogen debranching enzyme but only shared around 50% nucleotide identity (75.71% identify using Blosum62 matrix), which could affect the functionality of this protein. *L. gasseri* strains shared a more similar surface protein profile, 19/126 and 19/125 surface proteins were unique to Lga_V and Lga_I, respectively. Notably, *L. gasseri* is not a S-layer forming species. Lga_V had a unique bacteriocin lactacin Laf A gene compared to Lga_I. Lga_I contained unique proteins such as glutamine-binding protein GlnH.

Exopolysaccharides not only serve as a protective layer for bacterial survival during GI transit but also plays a pivotal role in the host interaction as they are known to have immune modulatory properties (Castro-Bravo et al., 2018). After a BLAST search against reference EPS cluster from Lcr_I and manual curation through individual genome, we found Lcr_V contained no EPS cluster while the rest of the three genomes contained complete but different EPS clusters sequence (Figure 1B). Genes associated with ESP production and polymerization such as *epsA-E* (located at the beginning of the cluster) and *epsJ* and *epsI* (located at the end of the cluster) shared nucleotide similarity (around 60%) but no similarity in the middle of the cluster (Figure 1B). It appeared that different glycosyltransferases were encoded between these three strains, indicating these EPS were made of different sugar monomers.

Human body sites are rich reservoirs of viral particles. We then investigated the presence of viral DNA in the genomes of the four *Lactobacillus* strains. Two possible intact prophage regions were detected in Lcr_V, one was 45.8 kb and the other one was 60.9 kb (Figure 1C). Two potential intact prophage regions were detected in Lga_V, one was 36.9 kb and the other one was 40.7 kb (Figure 1C). A questionable prophage region was detected in Lga_I and Lcr_I due to missing of some signature phage proteins such as capsid, head, fiber, and plate proteins (Figure 1C). We also screened the bacterial genomes for CRISPR-Cas system, the only known adaptive immune system of prokaryotes against phages, which can play a role in bacterial survival and colonization. Type II-A system was detected in Lcr_V, Lga_V and Lga_I, and Type I-E system was detected in Lga_I (Figure 1D). The signature Cas9 protein in Lcr_V was interrupted with a hypothetical protein, likely rendering it non-functional. Lcr_I contained a Type I-E system which had three separate CRISPR arrays. Lga_V and Lga_I shared very similar CRISPR locus, except Lga_I had almost double number of spacers as Lga_V.

### Phenotypic Assays Reveal Niche-Specific Adaptation

**FIGURE 2:**
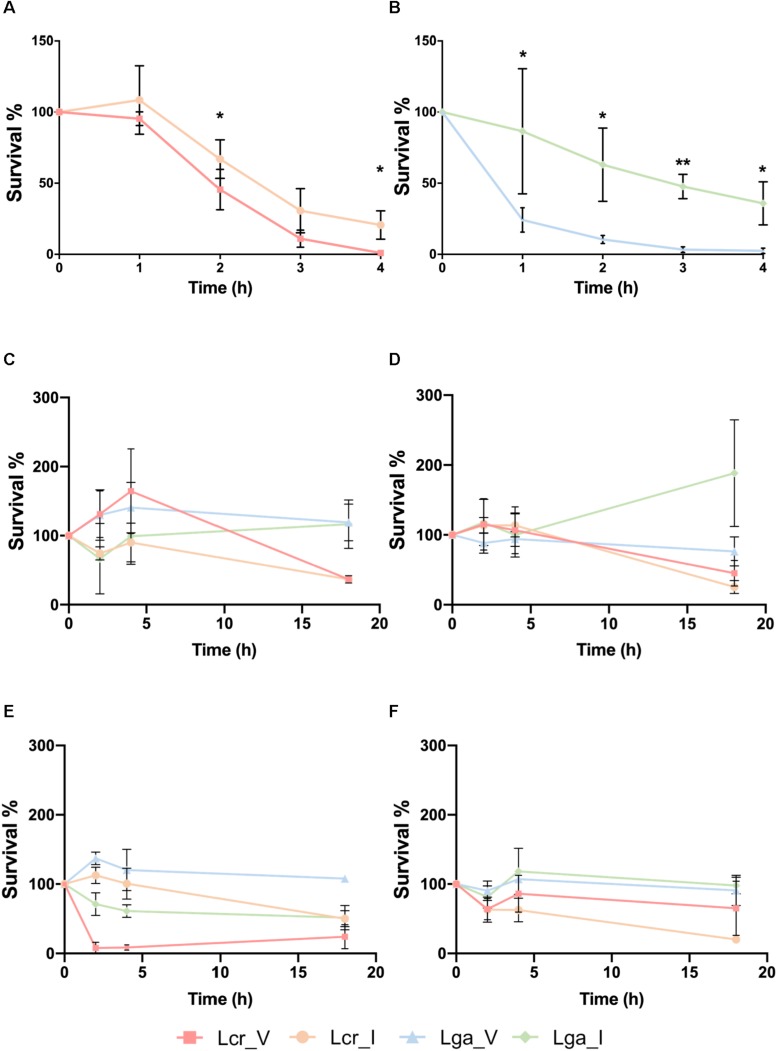
Stress challenges of the *Lactobacillus* strains. Survival of *Lactobacillus* strains in the small intestinal juice **(A,B)**. The *L. crispatus* **(A)** and *L. gasseri* **(B)** strains at stationary phase were challenged in the small intestinal juice for 4 h at 37°C under anaerobic condition. Survival of *Lactobacillus* strains exposed to acidified MRS broth (pH 4) with HCl at log phase **(C)** or stationary phase **(D)**, and survival of *Lactobacillus* strains exposed to acidified MRS (pH4) at log phase **(E)** or stationary phase **(F)**. The bacteria were incubated at 37°C anaerobically and sampled at 0, 2, 4, and 18 h. The data represents the means ± standard deviation of the means for three independent biological replicates. The survival rate within each species at each time point was compared for significant differences using *t*-test. ∗*p*-value < 0.05, ∗∗*p*-value < 0.01.

The vaginal intestinal isolates of both *L. gasseri* and *L. crispatus* above were used to assess potential niche-specific adaption and potential probiotic performance (Table 1). We first conducted a small intestine juice challenge assay to investigate whether the four strains vary in their ability to survive in simulated intestinal conditions. Both intestinal *L. crispatus* (Lcr_I) and intestinal *L. gasseri* (Lga_I) strains demonstrated higher recovery rate than the vaginal strains (Figures 2A,B). Survival rate in both vaginal *L. crispatus* (Lcr_V) and Lcr_I showed reduction over time with a significantly higher recovery rate for Lcr_I at 2h and 4h time point (Figure 2A). Only 24% of the vaginal *L. gasseri* (Lga_V) cells survived after one-hour-exposure to the small intestine juice, which was significantly lower than the recovery rate of Lga_I (86%) (Figure 2B). At the end of the 4-h-challenge, only 1% of Lcr_V cells and 3% of Lga_V were recovered while 21% of Lcr_I and 36% of Lga_I were recovered (Figures 2A,B).

The ability of the strains to survive in acidic conditions was tested in acidified MRS (pH4) with either lactic acid (representative acid from the vaginal tract) or hydrochloric acid (representative acid from the gastric juice) (Figures 2C–F). We observed that at log phase, Lcr_V was able to grow by 164% after two 2 h of hydrochloric acid exposure, which was not observed in the Lcr_I (Figure 2C). Similarly, Lga_V demonstrated higher resistance toward hydrochloric acids than Lga_I at log phase (Figure 2C). At stationary phase, the four *Lactobacillus* strains demonstrated comparable resistance to hydrochloric acid for the first 4 h (Figure 2D). Log phase Lga_V was more resistant to lactic acid than Lga_I. Interestingly, log phase Lcr_V was very sensitive to lactic acid which was not observed in Lcr_I (Figure 2E). At stationary phase, lactic acid resistance was observed to be different between species rather than within the same species (Figure 2F), with *L. gasseri* being more resistant than *L. crispatus*. Overall, the vaginal strains showed higher acid resistance than the intestinal strain within the same species, except when log phase *L. crispatus* was exposed to lactic acid, Lcr_V was significantly more sensitive than Lcr_I. We also observed that *L. gasseri* is a stronger lactic acid producer than *L. crispatus* which corresponds to the intrinsic higher acid resistance in *L. gasseri* (Supplementary Figure S1).

### Variation in Carbohydrate Utilization Between and Within Species

**FIGURE 3:**
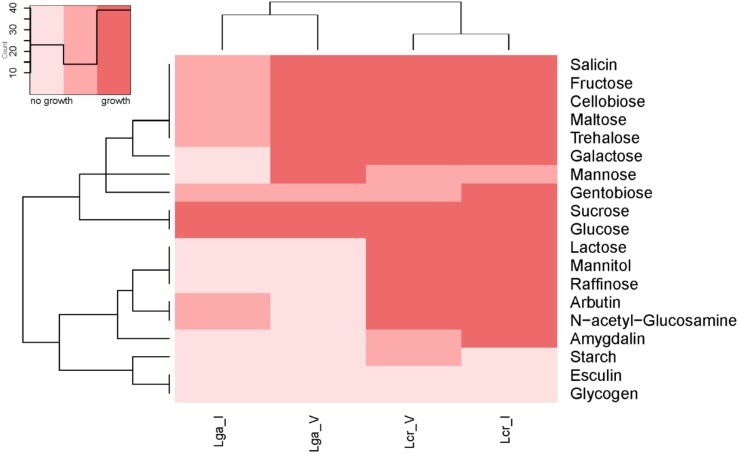
Heatmap of carbohydrate metabolism of *Lactobacillus* strains. The carbohydrate metabolism abilities of the *Lactobacillus* strains were evaluated using API test. Hierarchical clustering was performed based on both strains and type of carbohydrates.

**FIGURE 4:**
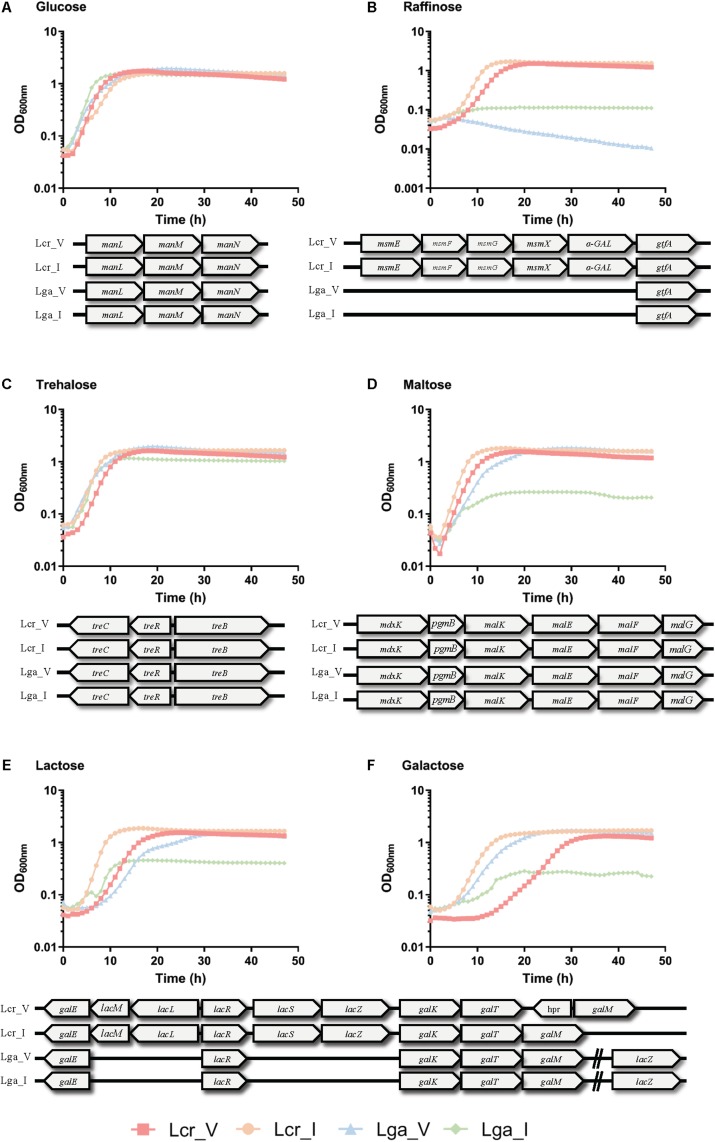
Growth curves of *Lactobacillus* strains in semi-defined medium (SDM). *Lactobacillus* strains were grown in SDM supplemented with 1% of glucose **(A)**, trehalose **(B)**, raffinose **(C)**, maltose **(D)**, lactose **(E)**, and galactose **(F)**. The corresponding sugar operon is shown below each growth curve. The data represents the mean of three biological replicates.

The carbohydrate utilization profile was assessed using the standard API strips (Figure 3). *L. crispatus* was able to metabolize a wider range of sugars than *L. gasseri* including raffinose, lactose, mannitol, arbutin, and *N*-acetylglucosamine. The two *L. crispatus* strains shared similar carbohydrate metabolism profile except Lcr_I was observed to ferment amygdalin and gentobiose better than Lcr_V. The two *L. gasseri* strains differed slightly in their abilities to ferment carbohydrates. Lga_V was able to ferment salicin, fructose, cellobiose, maltose, trehalose, galactose, and mannose better than Lga_I. On the other hand, Lga_I was able to grow on arbutin and *N*-acetylglucosamine. Interestingly, none of the strains was able to ferment glycogen. Growth in SDM supplemented with different sugars was then assessed, and the corresponding metabolic operons were predicted through *in silico* analyses for each strain (Figure 4). In this regard, both *L. gasseri* strains lacked the raffinose catabolic operon and consequently failed to grow in SDM supplemented with raffinose (Figure 4B). Lga_I showed little growth in SDM supplemented with maltose despite the presence of a putative maltose operon in its genome. Despite of some SNPs detected in the maltose operon of Lga_I compared to that of Lga_V which could render its functionality, high nucleotide percentage identity (99.7%) was shared between the two (Figure 4D). The Lga_I and Lga_V (to a lesser degree) struggled to ferment lactose and galactose which correlated with the absence of some key proteins such as *lacM*, *lacL*, and *lacS* in their genomes (Figures 4E,F). Lcr_I showed better growth in SDM supplemented with lactose (Figure 4E) or galactose (Figure 4F), which might be correlated with the minimal presence of lactose and galactose in the vaginal tract, contrast to a more abundant presence of galactose and glucose in the small intestine.

### Epithelium-Bacterium Interactions Are Strain Specific

**FIGURE 5:**
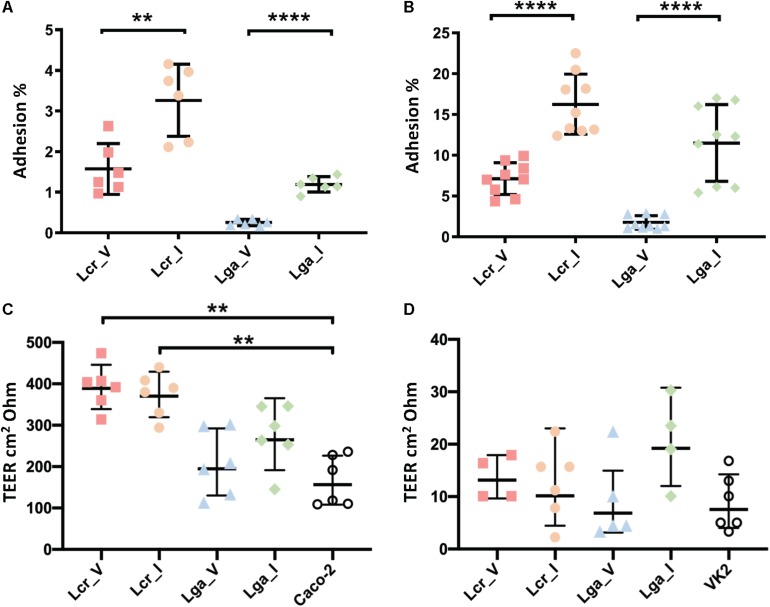
Interaction of *Lactobacillus* strains with Caco-2 and VK2 cell lines. Adherence **(A,B)** and barrier integrity analyses **(C,D)** were displayed as counts and transepithelial electrical resistance (TEER), respectively. The TEER data represents the change in TEER measurement over 24 h. The data represents the means ± standard errors for three independent biological replicates with technical replicates within each biological replicate. For the TEER test, one-way ANOVA and Tukey test were performed to see if the means were significantly different among groups. The statistical analyses were performed in R studio, v1.2.5001. ∗∗*p*-value < 0.01, ∗∗∗∗*p*-value < 0.0001.

Caco-2 colon cancer epithelial cell line and VK2 vaginal epithelial cell line were used to investigate bacterial-host interactions in terms of adherence and barrier integrity. Overall, *L. crispatus* demonstrated better adherence ability than *L. gasseri* to both Caco-2 cell line and vaginal VK2 cell line (Figures 5A,B). Intestinal strains showed significantly higher adherence than vaginal strains for both *L. crispatus* and *L. gasseri* and in both cell lines (Figures 5A,B). For the epithelial integrity assay, all four strains increased TEER compared to the bacteria free control in the Caco-2 cell line (Figure 5C), although the differences in *L. gasseri* strains were not significant. This was consistent with the two *L. crispatus* strains displaying higher adherence in both cell lines. Noticeably, *Lactobacillus* strains did not alter the TEER significantly after 24 h incubation in the VK2 cell line (Figure 5D). Generally, the TEER values obtained in VK2 cell line were very low compared to those obtained with Caco-2 cell line and this can be attributed to the different tissue morphology. We also visualized the cell morphology of each strain using SEM. It showed that *L. gasseri* strains had a smooth surface whereas *L. crispatus* had a rougher cell surface, potentially due to the production of exopolysaccharides or other surface proteins such as surface layer protein (Supplementary Figure S2), which may contribute to the differential interaction profile with the epithelial cell lines between the two species.

### Vaginal Isolates Performed Better in Simulated Vaginal Fluid

**FIGURE 6:**
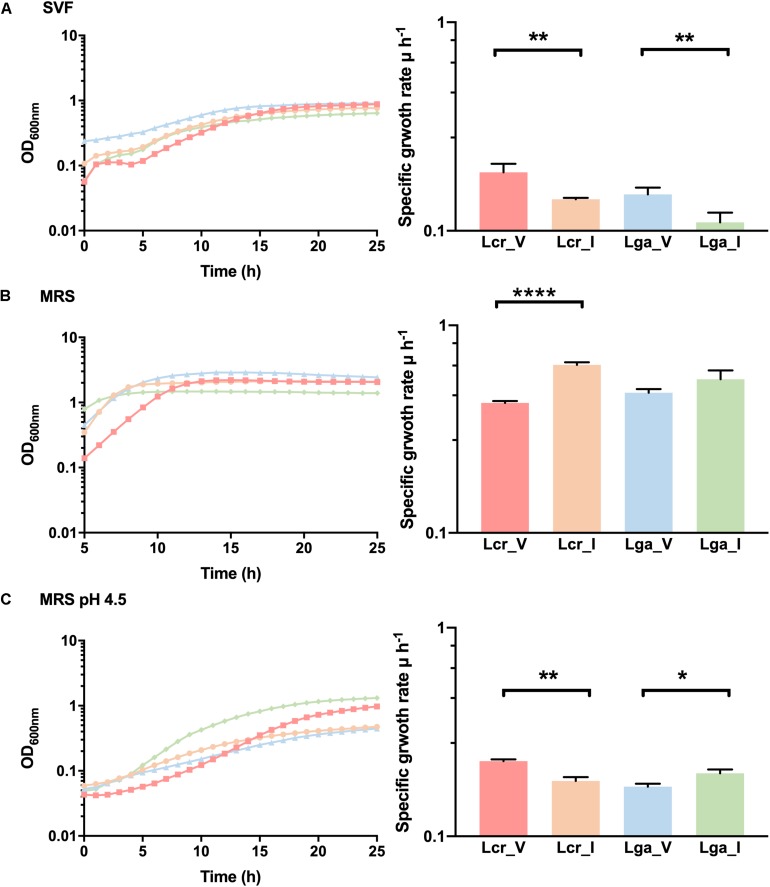
Growth curves and mid-log growth rate of *Lactobacillus* strains. The growth curves were performed in simulated vaginal fluid (SVF) **(A)**, MRS **(B)**, and lactic acid-acidified MRS at pH 4.5 **(C)**. The growth curves were performed in biological triplicates. The growth rate data represents the mean ± standard deviation for biological triplicates. ∗*p*-value < 0.05, ∗∗*p*-value < 0.01, ∗∗∗∗*p*-value < 0.0001.

A SVF (Table 2) that mimics the composition and the pH of the vaginal environment was developed to assess bacterial growth and potential niche-specific adaptation to the vaginal environment. Differential growth pattern was observed for the four strains in SVF compared to MRS (Figure 6). In SVF, the four strains showed extended lag phase compared to growth in MRS, indicating an initial adaptation was required prior to bacterial proliferation due to the limited nutrient availability and low pH in SVF (Figure 6A). The specific growth rate (μ) of Lcr_V, Lcr_I, Lga_V, Lga_I at mid-log phase in SVF was 0.19, 0.14, 0.15, and 0.11 h^–1^, respectively. Noticeably, both vaginal strains performed better than their corresponding intestinal strains in SVF. In MRS, Lcr_I had the highest specific growth rate of 0.64 h^–1^ at the mid-log phase, and Lcr_V had the longest lag phase and lower growth rate (μ = 0.42 h^–1^) compared to Lcr_I (Figure 6B). Although growth was significantly inhibited in lactic acid acidified MRS at pH4.5 across all four strains (Figure 6C), the growth pattern was different from that in SVF, indicating other components of SVF besides lactic acid, such as inadequate nutrients availability, may serve as growth limiting factors.

### RNA-Seq Analysis Revealed Condition-Specific Gene Expression

**FIGURE 7:**
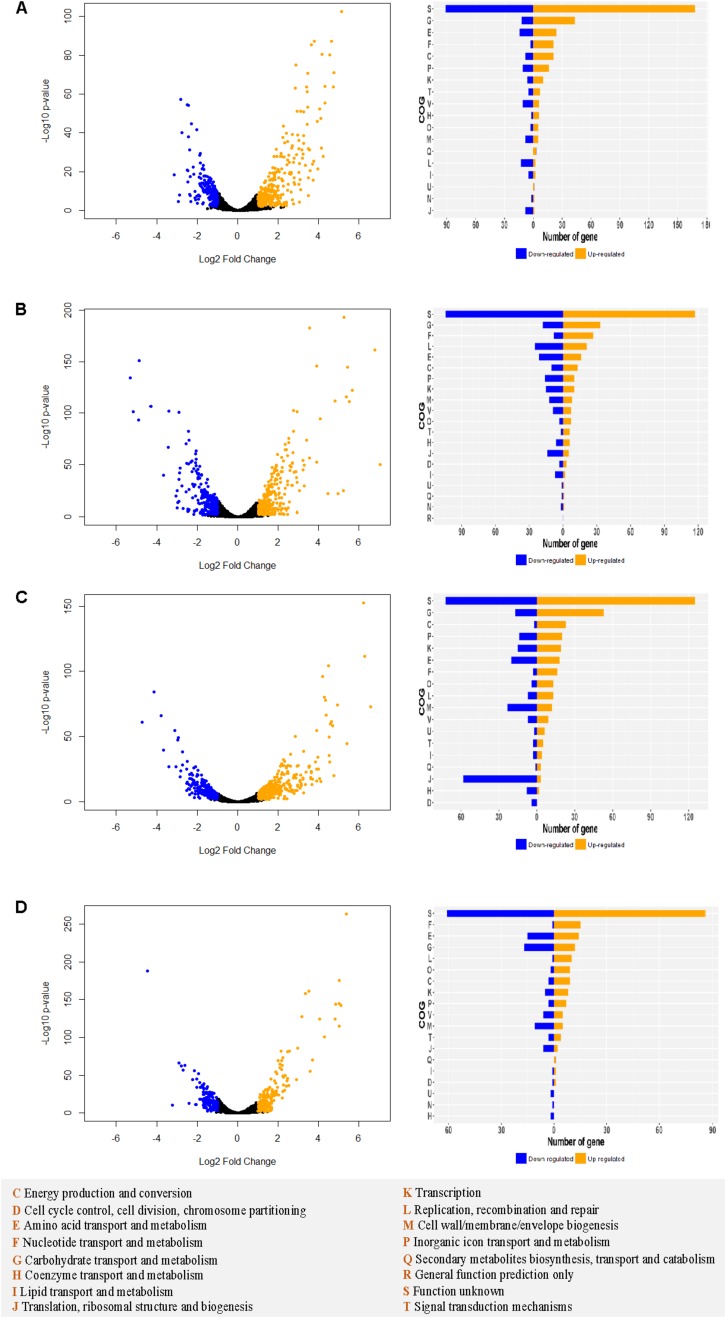
Differential transcriptomic analysis in MRS vs. SVF in *Lactobacillus* strains. For Lcr_V **(A)**, Lcr_I **(B)**, Lga_V **(C)**, and Lga_I **(D)**, the differentially expressed genes (| Log2 fold change ratio| ≥1 and *p*-value < 0.01) are labeled in blue (downregulated) or orange (upregulated) on the volcano plot (left panel). The differentially expressed genes for each strain are assigned to cluster of orthologous groups (COGs) (right panel). The COGs legend is provided at the bottom of the figure.

Transcriptomic analyses were performed in log phase cultures grown in SVF and MRS medium to investigate the mechanism underlying the observed differential growth among the strains. MRS served as a baseline of transcriptomic profile of the strains. Substantial gene expression differences between SVF and MRS were observed in all four strains (Supplementary Figure S3). Besides the functionally unknown proteins, the top categories of upregulated genes belong to carbohydrate transport and metabolism, amino acid transport and metabolism, nucleotide transport and metabolism, and energy production and conversion (Figure 7). Vaginal strains had more significantly upregulated genes (Log2 ratio ≥ 1 and *p*-value < 0.01) in different functional categories such as carbohydrate transport and metabolism, than the intestinal strains in both *L. crispatus* and *L. gasseri* overall.

**FIGURE 8:**
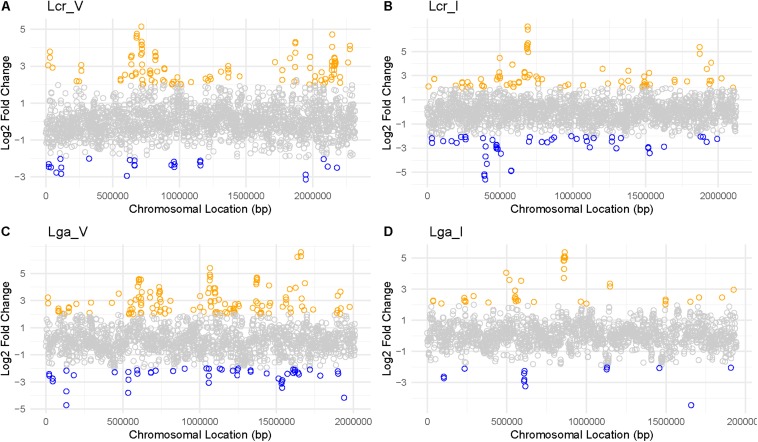
Log2 fold change ratio mapped to each genome of *Lactobacillus* strains. For Lcr_V **(A)**, Lcr_I **(B)**, Lga_V **(C)**, and Lga_I **(D)**, the log2 ratio was mapped to the chromosomal location to reveal any differential gene expression hot spots. Highly differentially expressed genes (|Log2 fold change ratio| ≥2 and *p*-value < 0.01) were labeled in blue (downregulated) or orange (upregulated).

Mapping the Log2 differential expression ratio against the chromosomal location reveals transcriptomic regulation hot spots and operons (Figure 8 and Supplementary Table S1) with genes involved in nutrient availability and stress response for SVF. One up-regulated region occurred at 750,000 bp and 2,000,000 bp in both vaginal and intestinal *L. crispatus* strains (Figures 8A,B), while Lcr_V had notably more up-regulated genes than Lcr_I at 2,000,000 bp region. Lga_V had multiple up-regulated regions around 650,000 bp, 1,000,000 bp, and around 1,500,000 bp regions (Figure 8C). Conversely, Lga_I showed less differential gene regulation overall, with no apparent hot spot except near 750,000 bp region (Figure 8D). Many of these operons had functions involved in carbohydrate metabolism, energy production, amino acid metabolism and nucleotide acid metabolism (Supplementary Table S1).

**FIGURE 9:**
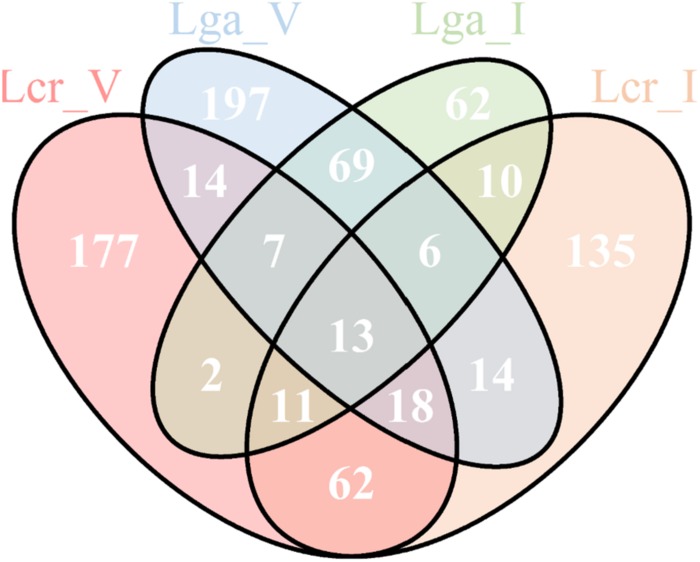
Upregulated genes in simulated vaginal fluid (SVF) across four *Lactobacillus* strains. The Venn diagram presentation of the differentially upregulated genes in SVF (Log2 fold change ratio ≥1 and *p*-value < 0.01) of each strain based on protein ortholog search.

A total of 13 common genes were found to be upregulated across the four strains (Figure 9 and Supplementary File S2). Among them, the entire pyrimidine synthesis operon was upregulated along with other genes such as *dps*, a DNA protection protein, *IIdD*, a L-lactate dehydrogenase, and an acid shock protein. There were 177 upregulated genes specific to Lcr_V including *cop*A which codes for a copper-exporting P-type ATPase, *ger*N which codes for a Na(+)/H(+)−K(+) antiporter, and other genes related to energy conservation and production. Interestingly, Lga_V and Lcr_V had the most unique upregulated genes compared to the two intestinal strains, which might indicate the upregulation of genes specific to vaginal adaptation (Supplementary File S2).

## Discussion

The health-promoting beneficial properties of indigenous lactobacilli vary among species and human niches. Whether the strains of the same species isolated from two distinct environments, such as GI tract and vaginal tract, are functionally different is a key question to answer in the probiotic industry. This knowledge is critical to screen, select and formulate probiotics for vaginal health vs. intestinal applications. Presumably, some features are strain dependent while others are inherent to the different environmental conditions. Here, we compared *L. gasseri* and *L. crispatus* intestinal and vaginal isolates to determine their core and unique functional features, and investigate whether they have functional differences reflecting the unique attributes of the vaginal vs. intestinal environment.

Niche-specific features have been previously detected in probiotic species and strains through comparative genomic analyses. In this regard, O’Sullivan et al. (2009). demonstrated that dairy-derived lactic acid bacteria (LAB) were more likely to have genes coding for proteolytic system and restriction endonuclease, and gut-derived LAB tend to have genes coding for sugar metabolism and BSH, reflecting also niche adaption to milk or gut conditions. Petrova et al. (2018) detected a *spaCBA* locus (associated with pili-mediated intestinal epithelial adhesion) in *L. rhamnosus* GG, isolated from human feces, but absent in *L. rhamnosus* GR-1 (female urethra isolate) and LC705 (dairy product isolate). In addition, *L. rhamnosus* GR-1 produced a different exopolysaccharide than the other two strains which was associated with better oxidative stress resistance, a phenotype highly relevant in vaginal condition. Mining of EPS gene clusters in the four *Lactobacillus* strains in the present study revealed that Lcr_V did not contain an EPS cluster. The other three strains harbor an EPS cluster with different key genes reflecting the potential production of EPS with different monosaccharide composition. The different EPS production profile predicted by our *in silico* analysis could lead to different host interaction among the four strains. Additionally, the ability to produce exopolysaccharides is associated with potential benefits of protection and persistence in the gut environment (Castro-Bravo et al., 2018). To verify the *in silico* analysis of EPS cluster of the four strains, further EPS isolation and characterization would be required. The *in silico* surface protein analysis suggested surface protein composition was different both within and between species. Mendes-Soares et al. (2014) reported that certain COGs involved in phosphate transport and hydrolases were present exclusively in the vaginal *Lactobacillus* species, which may indicate potential mechanism for their specialization to the vaginal environment. Similarly, we discovered that Lcr_V encoded a unique phosphate-import permease protein PhnE. Likewise, prophages may play an important, yet still unknown role in microbiome synergies. Up to 20% of bacterial genome can be constituted of viral DNA which contribute significantly to species and strain diversity (Canchaya et al., 2004). Cryptic prophages in *Escherichia coli* K-12 has been demonstrated to confer multiple benefits to the host including resistance to osmotic stress, biofilm formation, quinolone and beta-lactam antibiotic resistance (Wang et al., 2010). It has been reported that prophages in *Lactobacillus reuteri* can be activated during GI transit, particularly in a fructose-enriched diet (Oh et al., 2019a). It was also reported that although prophages in *L. reuteri* 6475 were associated with a fitness trade-off, they can be advantageous in its intestinal niche by killing its competitors through generating active phages (Oh et al., 2019b). The different predicted prophage regions in the four *Lactobacillus* strains in our study likely reflect the different viral communities between human GI and vaginal tract. CRISPR-Cas system is the only known adaptive immune system in prokaryotes to combat phage attacks. Protection and persistence in the community can be conferred by the possession of functional CRISPR-Cas system. Lcr_I contained a Type I-E CRISPR system whose functionality has been validated in our previous work (Hidalgo-Cantabrana et al., 2019). The Type II-A system in Lcr_V is likely unfunctional due to the interruption of Cas9 protein by a hypothetical protein. Likewise, the functionality of Type II-A system in Lga_V and Lga_I has also been verified previously (Stout et al., 2018).

We observed both niche-specific and strain-specific variation between the four *Lactobacillus* strains in the phenotypic assays. Both intestinal strains demonstrated significantly higher resistance to small intestinal juice than the vaginal strains within in the same species. Interestingly, vaginal strains showed higher HCl resistance but similar lactic acid resistance compared to the intestinal strains. Lactic acid resistance in Lcr_V increased significantly from log phase to stationary phase, indicating the organic acid resistance mechanisms are turned on at the later stage of growth cycle. Intestinal strains showed higher adhesion rate to both intestinal and vaginal epithelial cells, and demonstrated better ability in improving barrier integrity in the Caco-2 cell line but not VK2 cell line (Figure 5). However, it remains controversial whether bacterial colonization ability is equally important for the commensal microbiota living in the vaginal and intestinal environment. In order to compete with the higher diversity detected in the gut microbiome and to withstand the flow movement due to peristalsis during digestion (Sanderson, 1999), intestinal bacteria may evolve to develop stronger colonizing ability than vaginal strains. While Caco-2 is a well-established cell culture model to study bacterial adherence and epithelial integrity (Li et al., 2004; Muller et al., 2009; Valenzano et al., 2015), our results suggest that the VK2 cell line might not an ideal model for studying the bacterial influence on tight junction proteins in vaginal tissues. While there is an abundance of intercellular junction proteins in the basal and supra-basal epithelial layer in the vaginal tissues, the superficial layers of stratified squamous epithelial cells lack tight junction proteins, making this region a semi-permeable microenvironment (Blaskewicz et al., 2011). Failure to detect a significant increase in TEER measurement in VK2 cell lines might result from the lack of direct interaction between the probiotics and the junction proteins which are only expressed in the deeper basal epithelial layers.

Vaginal fluid is a complex biological fluid, composed of water (up to 95%), proteins such as albumin, mucins, and immunoglobulins, carbohydrates such as glycogen (around 15 g/L), glucose (between 6.2 and 10 g/L), and trace amount of maltose, mannose, and glucosamine, salts such as Na^+^, K^+^, and Cl^–^, urea, and other molecules (Wagner and Levin, 1980; Rajan et al., 1999; Valore et al., 2002; Dasari et al., 2007). A variety of organic acids such as lactic acid, acetic acid, succinic acid, and propionic acid, has been identified in the vaginal environment, with lactic acid being the major acid (Boskey et al., 2001). While some published studies investigating vaginal fluids focused on drug delivery and diffusion properties (Rastogi et al., 2016; Tietz and Klein, 2018), relatively few studies investigated bacterial growth (Juárez Tomás and Nader-Macías, 2007), and some do not account for the relevant acidic vaginal pH (Geshnizgani and Onderdonk, 1992; Owen and Katz, 1999). The SVF presented in this work has a pH of 4.5 and components that represent the actual vaginal fluid. Although the actual growth difference between vaginal and intestinal strain is small, the vaginal strains had significantly higher growth rate and shorter lag phase, indicating better adaptation to the SVF environment. This was especially remarkable in the case of Lcr_V, which was the slowest grower in MRS but the fastest one in SVF displaying clear adaptation to SVF which simulates vaginal condition.

Using MRS as a standard medium control, differential transcriptomic analysis of the *Lactobacillus* strains grown in SVF revealed dramatic changes in gene expression profile. Multiple carbohydrate metabolism and transport systems such as maltose phosphorylase, glucan 1,6-alpha-glucosidase, and trehalose transporter ATP-binding protein SugC were upregulated in SVF. It is possible that glycogen was partially hydrolyzed by α-amylase to derive short-chain carbohydrates that can be further metabolized by the *Lactobacillus* strains (Spear et al., 2014). It also showed that maintenance of redox balance and energy conservation appeared to be crucial for growth in SVF. For example, fumarate hydratase class II, commonly upregulated for all strains in SVF, not only participates in citric acid cycle during aerobic growth but also takes part in the reductive side of the anaerobic cycle where it uses fumarate as an anaerobic electron acceptor to restore reducing power (Woods et al., 1988). Among the genes that were commonly upregulated across all the four strains, the entire pyrimidine biosynthetic pathway was upregulated indicating that pyrimidine was lacking in SVF, which could lead to insufficient production of fundamental nucleotides such as cytosine, uracil and thymine. As expected, genes related to stress responses were also upregulated. The *dps*, which encodes a DNA protection protein during starvation was also upregulated across all four strains. This iron-binding protein was known to provide oxidative stress resistance by preventing formation of hydroxyl radicals through Fenton reaction in *Campylobacter jejuni* and acid and alkaline shock in *E. coli* (Ishikawa et al., 2003; Nair and Finkel, 2004).

Overall, this study demonstrated that the isolation source of a strain could influence its potential probiotic functionality, and should be taken into account for probiotic formulation. Some of our results could be strain-specific rather than isolation source-dependent given that we only investigated two strains from each species for the analyses due to limitation of resources and practical issues. Although SVF aimed to resemble the actual vaginal fluid, it is still significantly different from *in vivo* vaginal environments, lacking the microbiota, the epithelial tissue and other hormonal influences. It would be interesting to include a medium that resembles the intestinal fluid for the differential transcriptomic analysis. However, the current small intestinal juice model does not support bacterial growth, which renders it inappropriate for comparison with transcriptomic profile in SVF. Nonetheless, the functional characterization of *L. crispatus* and *L. gasseri* intestinal and vaginal strains in this study demonstrated potential niche-specific adaptation along with strain-specific features. This study also highlights the need to further characterize the common and unique functional properties of vaginal and intestinal probiotics, preferably in conditions that mimic the nutrient availability, pH, human tissue represented in the *in vivo* conditions. These findings should guide the rational formulation of next-generation probiotics for intestinal and vaginal health.

## Data Availability Statement

The datasets generated for this study can be found in the NCBI ID PRJNA546252, SAMN11958879–SAMN1195889, ACK R00000000, SGWL00000000, ACGO00000000, and CP043924.

## Author Contributions

MP designed the study, performed *in silico* analyses, carried out the experiments, and wrote the manuscript. CH-C and RB designed the study and provided supervision and support. YG and RS-D designed the study and provided technical input. All authors contributed to the manuscript editing and approved the final version.

## Conflict of Interest

RB and CH-C are inventors on several patents related to probiotics and their uses. RB is a shareholder of Caribou Biosciences, Intellia Therapeutics, Locus Biosciences, and Inari Agriculture, and a co-founder of Intellia Therapeutics and Locus Biosciences. CH-C, an advisor and co-founder of Microviable Therapeutics. The remaining authors declare that the research was conducted in the absence of any commercial or financial relationships that could be construed as a potential conflict of interest.

## Footnotes

1 http://www.bioinformatics.babraham.ac.uk/projects/fastqc/

## Supplementary Material

FILE S1 SignalP predicted surface proteins for the four *Lactobacillus* strains. The orange highlighted proteins were unique predicted surface protein within each species.

FILE S2 Upregulated genes (Log2 ≥ 1 and *p*-value < 0.01) in SVF compared to growth in MRS for the four *Lactobacillus* strains.
