# Supplementary figures and images for: Comparative Analysis of Lactobacillus gasseri and Lactobacillus crispatus Isolated From Human Urogenital and Gastrointestinal Tracts
Source: Front Microbiol. 2020 Jan 22;10:3146. doi: 10.3389/fmicb.2019.03146 (PMC6988505; doi:10.3389/fmicb.2019.03146)

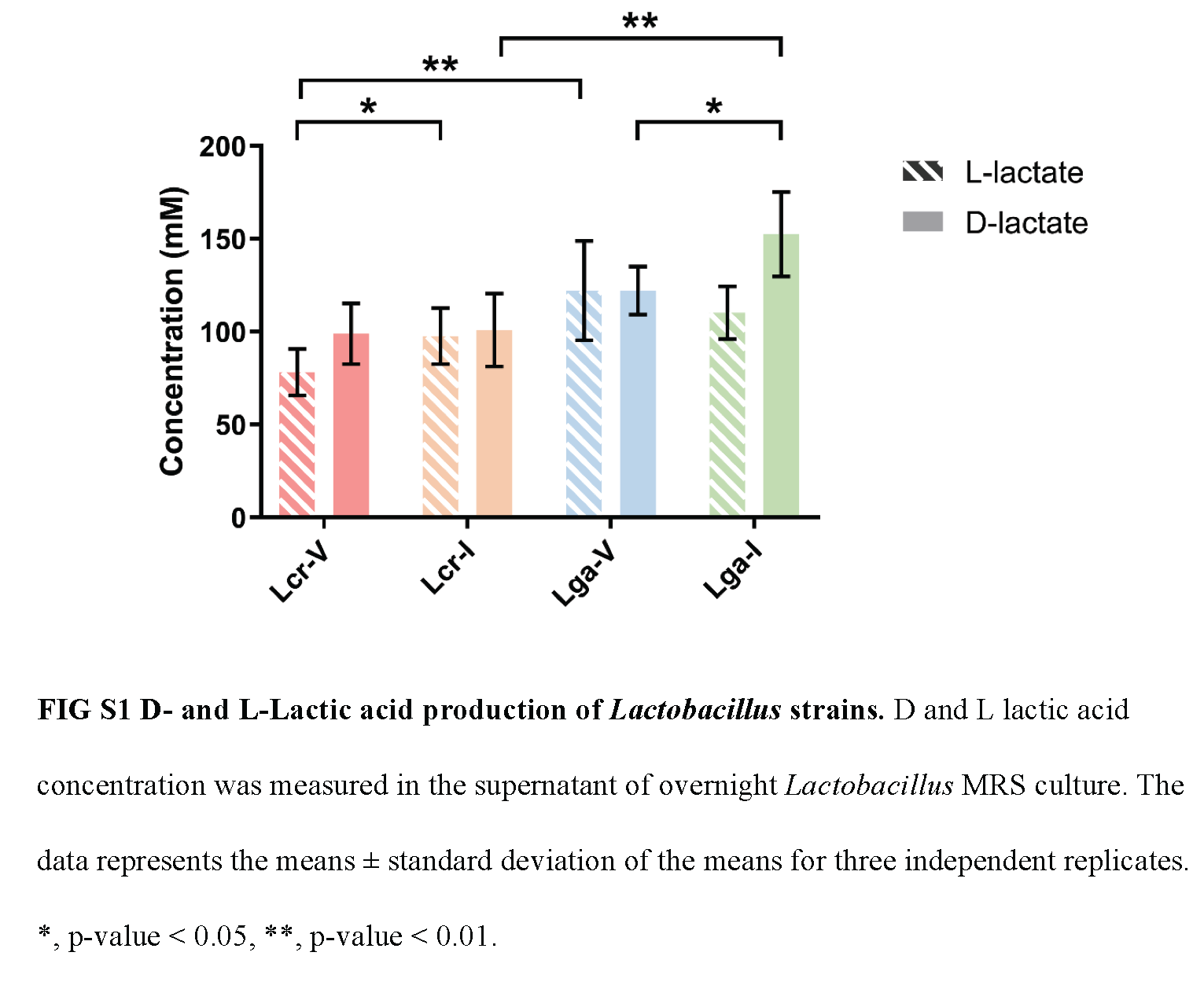

Supplement: Supplementary file 3 [file Image_1.TIFF]

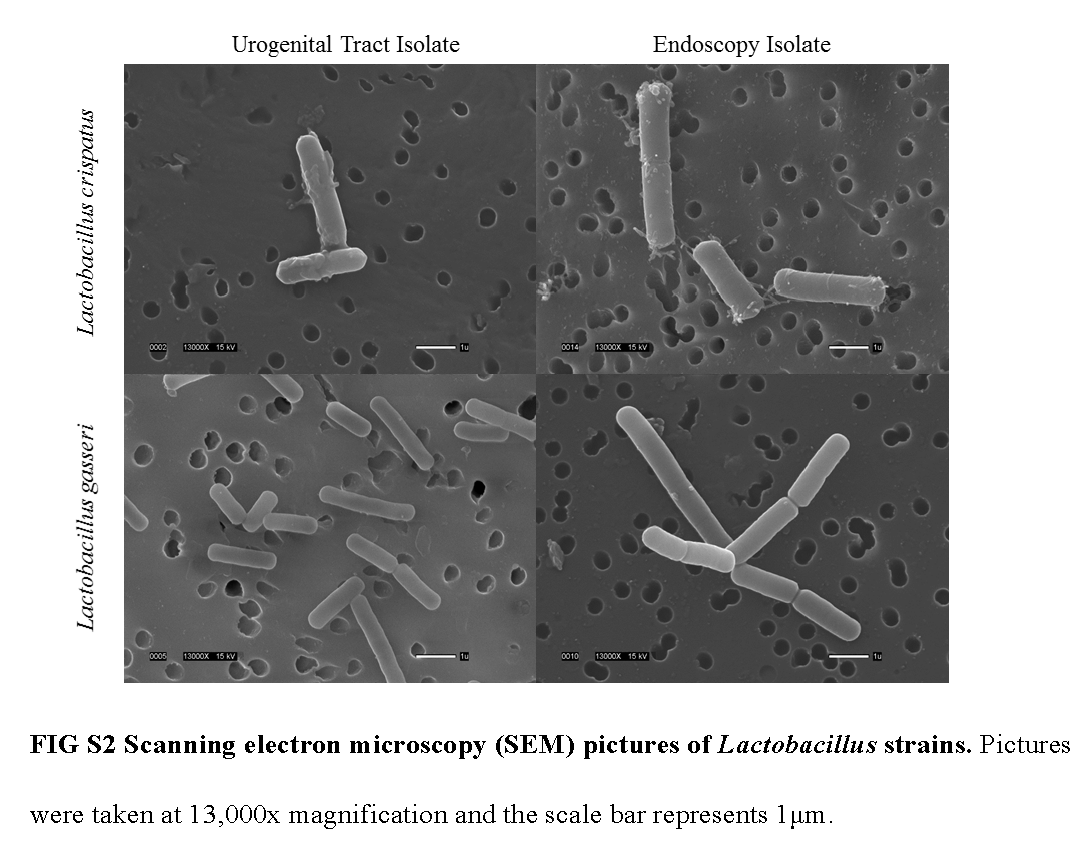

Supplement: Supplementary file 4 [file Image_2.TIFF]

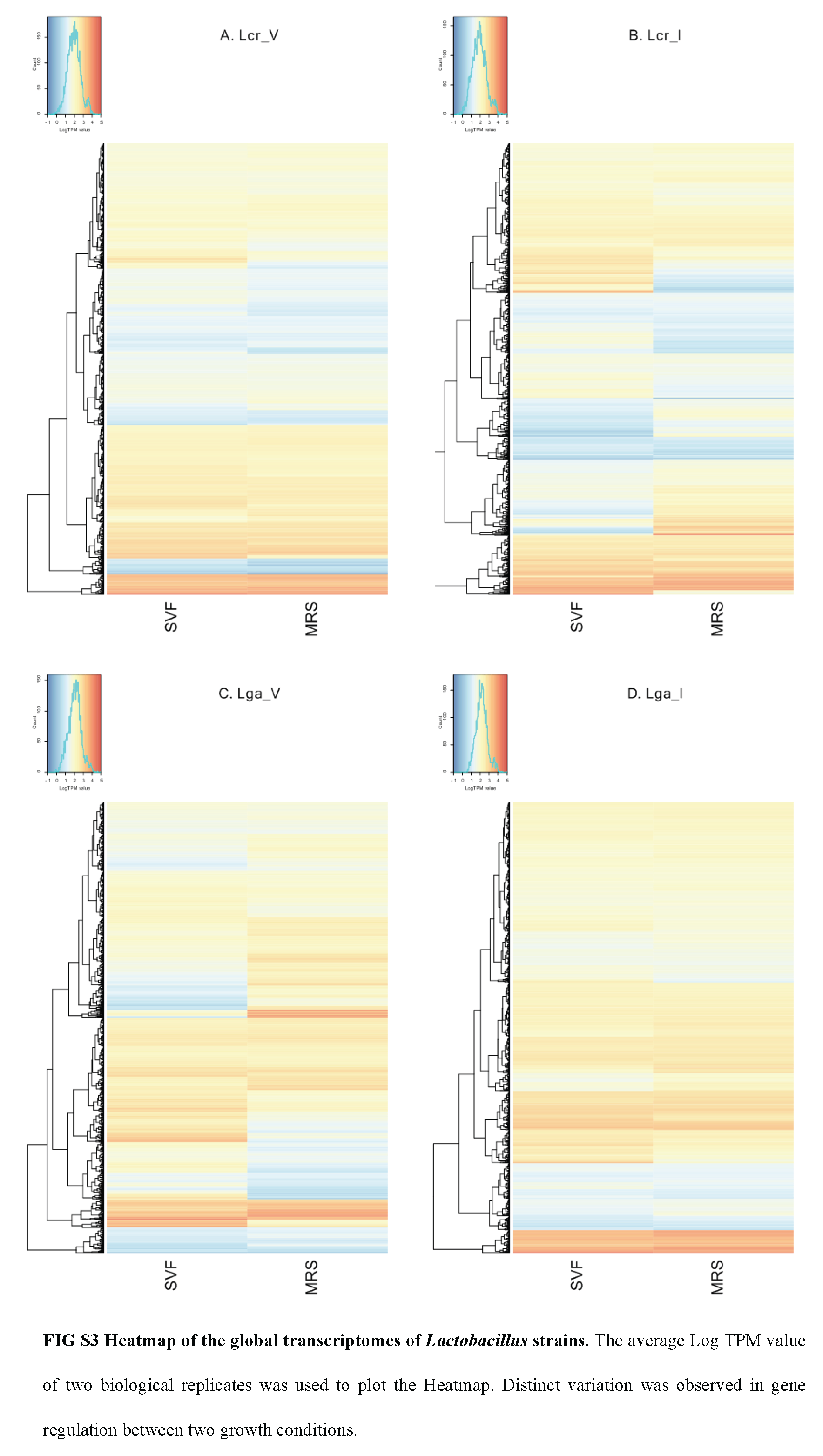

Supplement: Supplementary file 5 [file Image_3.TIFF]
