# Supplementary material for: Comparative Analysis of Lactobacillus gasseri and Lactobacillus crispatus Isolated From Human Urogenital and Gastrointestinal Tracts
Source: Front Microbiol. 2020 Jan 22;10:3146. doi: 10.3389/fmicb.2019.03146 (PMC6988505; doi:10.3389/fmicb.2019.03146)
Supplement: Supplementary file 6 [file Table_1.pdf]

TABLE S1 Upregulated operons in *Lactobacillus* strains grown in simulated vaginal fluid (SVF). The gene names and corresponding functions of the operon are listed.

Strain	Operon	Genes	Function
Lcr_V	1	Hypothetical protein, *gerN 2, sgIT, axeA, cah, nanE, nagA 2*	Amino acid metabolism
	2	*spuD, potA 1, ydcV 1*	Energy production/conservation
	3	*ywaC, czcR, tcrA, baeS 1*	Stringent response
	4	*pyrB, pyrC, carA 1, carB 1*	Pyrimidine synthesis
	5	*ribD, ribE, ribBA, ribH*	Riboflavin synthesis
	6	*mtlD, mtlF, mtlA*	Mannitol metabolism
	7	*phoU 2, pstB3 3, pstA2, pstC, pstS1 2*	Phosphate metabolism
	8	*sugC 3, yvdM, malP 2, bbmA, malL*	Maltose metabolism
Lcr_I	1	*pyrR, pyrB, pyrC, carA, carB*	Pyrimidine synthesis
	2	*ywac, dltR, spts*	Stringent response
	3	*ribH, ribBA, ribE, ribD*	Riboflavin synthesis
	4	*uraA, pyrR*	Uracil metabolism
Lga_V	1	*bglA 1, bglG, bglF*	Energy production/conservation
	2	*nplt, map2, pgmb, msmk, malG, dexB*	Carbohydrate metabolism
	3	*ade, potD, potA, potB,*	Energy production/conservation
	4	*pyrB, pyrC, carA, carB, upp*	Pyrimidine synthesis
	5	*Flp, ylbN, copZ, had*	Amino acid metabolism
	6	*rtpR*, membrane protein, *yvqK*	Energy production
	7	*yvgN, ykgC*	Pyridine metabolism
	8	*lacT, lacE, lacG, lacF*	Carbohydrate metabolism
Lga_I	1	*pyrDB, pyrR 1, pyrB, pyrC, carA, carB, pyrR 2*	Pyrimidine synthesis
	2	*adeC, spuD*	Nucleotide acid metabolism
	3	Hypothetical protein, *pepC, gadC*	Amino acid metabolism
